# Benserazide, a cystathionine beta-synthase (CBS) inhibitor, potentially enhances the anticancer effects of paclitaxel via inhibiting the S-sulfhydration of SIRT1 and the HIF1-α/VEGF pathway

**DOI:** 10.3389/fphar.2024.1404532

**Published:** 2024-05-17

**Authors:** Wei Zhao, Shasha Feng, Jian Wang, Zhenshuai Zhang, Lu Chen, Li Jiang, Ming Li, Tianxiao Wang

**Affiliations:** School of Pharmacy, Henan University, Kaifeng, Henan, China

**Keywords:** benserazide, paclitaxel, SIRT1, HIF1-α, VEGF, cancer

## Abstract

Cancer targeted therapy is essential to minimize damage to normal cells and improve treatment outcomes. The elevated activity of Cystathionine beta-synthase (CBS), an enzyme responsible for producing endogenous hydrogen sulfide (H_2_S), plays a significant role in promoting tumor growth, invasiveness, and metastatic potential. Consequently, the selective inhibition of CBS could represent a promising therapeutic strategy for cancer. Currently, there is much interest in combining paclitaxel with other drugs for cancer treatment. This study aimed to investigate the efficacy of combining benserazide, a CBS inhibitor, with paclitaxel in treating tumors. Firstly, we demonstrated CBS is indeed involved in the progression of multiple cancers. Then it was observed that the total binding free energy between the protein and the small molecule is −98.241 kJ/mol. The release of H_2_S in the group treated with 100 μM benserazide was reduced by approximately 90% compared to the negative control, and the thermal denaturation curve of the complex protein shifted to the right, suggesting that benserazide binds to and blocks the CBS protein. Next, it was found that compared to paclitaxel monotherapy, the combination of benserazide with paclitaxel demonstrated stronger antitumor activity in KYSE450, A549, and HCT8 cells, accompanied by reduced cell viability, cell migration and invasion, as well as diminished angiogenic and lymphangiogenic capabilities. *In vivo* studies showed that the combined administration of benserazide and paclitaxel significantly reduced the volume and weight of axillary lymph nodes in comparison to the control group and single administration group. Further mechanistic studies revealed that the combination of benserazide and paclitaxel significantly suppressed the S-sulfhydration of SIRT1 protein, thereby inhibiting the expression of SIRT1 protein and activating SIRT1 downstream Notch1/Hes1 signaling pathway in KYSE450, A549, and HCT8 cells. Meanwhile, we observed that benserazide combined with paclitaxel induced a more significant downregulation of HIF-1α, VEGF-A, VEGF-C, and VEGF-D proteins expression levels in KYSE450, A549, and HCT8 cells compared to paclitaxel alone. These findings indicated that benserazide enhances the anticancer effects of paclitaxel via inhibiting the S-sulfhydration of SIRT1 and down-regulating HIF-1α/VEGF signaling pathway. This study suggests that benserazide may have potential as a chemosensitizer in cancer treatment.

## 1 Introduction

Cancer incidence and mortality are increasing in China, making it a global cancer burden hotspot. Challenges like low early diagnosis rates and drug resistance hinder effective treatment. The need to enhance the effectiveness of chemotherapy is prompting researchers to explore innovative approaches like targeted therapy, thus providing new hope for cancer patients.

|Cystathionine beta-synthase (CBS) is an enzyme that catalyzes the synthesis of H_2_S from homocysteine and cysteine (Conter et al., 2020). H_2_S is a gasotransmitter that plays important roles in various physiological processes, including regulation of blood pressure, cardiovascular function, and inflammation (Han et al., 2019). In recent years, there has been growing interest in the role of CBS and H_2_S in cancer development and progression. Studies have shown that the elevated activity of CBS plays a significant role in promoting tumor growth, invasiveness, and metastatic potential (Youness et al., 2020; Padmanabhan et al., 2021; Santos et al., 2023). Consequently, the targeted inhibition of CBS could emerge as a promising therapeutic approach for cancer intervention.

Benserazide is a drug that inhibits the peripheral dopamine decarboxylase enzyme, which is used in combination with levodopa to treat Parkinson’s disease and Parkinson’s syndrome (Baba et al., 2022; Yang et al., 2023). Recently, it has been discovered that benserazide can also inhibit the activity of CBS protease by binding to pyridoxal phosphate (PLP), a coenzyme of the CBS protein (Druzhyna et al., 2016). Studies have shown that benserazide has inhibitory activity on both HCT116 cells and drug-resistant cells with high expression of CBS (Li et al., 2017; Untereiner et al., 2018).

Paclitaxel is a natural anticancer drug that has been shown to be effective against various types of cancer. However, the development of resistance to paclitaxel and its toxic side effects have limited its effectiveness when used alone (Abu et al., 2019; Smith et al., 2022; Hashemi et al., 2023). Currently, there is a lot of interest in combining paclitaxel with other drugs for cancer treatment (Yu et al., 2020; Xu et al., 2021; Škubník et al., 2023). This combination therapy approach not only helps to reduce the toxic side effects of chemotherapy but also allows for the reduction of the dosage of traditional chemotherapy drugs. Additionally, this approach can improve the overall curative effect of cancer treatment.

The purpose of this study was to investigate the potential benefits of using a combination of benserazide and paclitaxel in tumor treatment. The methodologies included the assessment of benserazide as a CBS inhibitor using advanced techniques like molecular docking (MD) and CETSA. Additionally, an exhaustive examination of the synergistic effects of the benserazide and paclitaxel combination was conducted through *in vitro* studies on antitumor activity and *in vivo* research on the inhibition of tumor lymph node metastasis, with the goal of gathering relevant data to guide future therapeutic strategies that may improve patient prognosis.

## 2 Materials

### 2.1 Cell lines

Human esophageal squamous cell line KYSE450 was donated by Xinxiang Medical College (Henan Province, China). Human lung cancer cell line A549 was a gift from Professor Li Hong at Henan University. Human colon cancer cell line HCT8 was donated by The Third Military Medical University (Chongqing, China). Human vascular endothelial cell (HUVEC) was generously donated by Shanghai University (Shanghai, China). Human lymphatic endothelial cells (HLEC) were purchased from Shanghai Binsui Biotechnology Co., Ltd. (Shanghai, China). The cells were cultured in RPMI-1640 medium (Gibco, Grand Island, New York, United States) supplemented with 10% fetal bovine serum (FBS; Zeta Life, Inc., San Francisco, CA, United States) at 37°C 5% CO_2_. All of cells were authenticated by STR.

### 2.2 Drugs

Benserazide (CAS: 14919-77-8) was procured from Selleck Chemicals LLC (Houston, Texas, United States), while Paclitaxel (CAS: MB1178-1) was sourced from Dalian Meilun Biotechnology Co., Ltd. (Dalian, Liaoning, China). For cell experiments, paclitaxel is dissolved in DMSO (Dimethyl sulfoxide), while benserazide is dissolved in PBS (Phosphate Buffer Saline). In animal experiments, paclitaxel is administered in saline with 1% Tween 80, and benserazide is administered in saline. The dosages and preparations of the drugs were based on references (Hu et al., 2020; Zhou et al., 2020).

## 3 Methods

### 3.1 Investigation into the binding affinity of benserazide with CBS protein

#### 3.1.1 Molecular docking (MD)

Molecular docking uses the 3D structure of CBS protein in the RCSB database, obtains the sdf structure of Benserazide through the Pubchem database, and converts it into a PDB file using OpenBabel. AutoDock Tools 1.5.6 software was used to dehydrate and hydrogenate protein targets, and the active ingredient and target protein formats were converted to pdbqt format. Finally, it is docked through AutoDock Vina. The molecular dynamics (MD) simulations were carried out by GROMACS 2020.3 software. The amber99sb-ildn force field and the general Amber force field (GAFF) were used to generate the parameter and topology of proteins and ligands, respectively. The simulation box size was optimized with the distance between each atom of the protein and the box greater than 1.0 nm. Then, fill the box with water molecules based on a density of 1. To make the simulation system electrically neutral, the water molecules were replaced with Cl- and Na + ions. Following the steepest descent method, energy optimization of 5.0 × 104 steps was performed to minimize the energy consumption of the entire system, and finally to reduce the unreasonable contact or atom overlap in the entire system. After energy minimization, first-phase equilibration was performed with the NVT ensemble at 300 K for 100 ps to stabilize the temperature of the system. Second-phase equilibration was simulated with the NPT ensemble at 1 bar and 100 ps. The primary objective of the simulation is to optimize the interaction between the target protein and the solvent and ions so that the simulation system is fully pre-equilibrated. All MD simulations were performed for 50 ns under an isothermal and isostatic ensemble with a temperature of 300 K and a pressure of 1 atm.

#### 3.1.2 CBS recombinant protein activity assay

CBS recombinant protein activity assay was designed to detect the inhibitory activity of benserazide on CBS protein. The following is a brief description of the method for detecting the activity of CBS recombinant protein. First, different concentrations of benserazide (14919-77-8, Selleck Chemicals LLC, Houston, Texas, United States) and the positive control aminooxyacetic acid (AOAA) (C13408-1G, sigma-aldrich, St. Louis, Missouri, United States) were added to a 96-well plate. Then, 50 μL of active buffer containing human recombinant CBS protein (5μg/well, Abt-P-1006, Beijing Ambition Biotechnology Co., Ltd., Beijing, China), 50 mM Tris HCl (pH8.0), L-cysteine (2 mM final concentration) (C0012, Solarbio Science & Technology Co.,Ltd., Beijing, China) and 5′-phospho pyridoxal 5′-phosphate (PLP) (5 mM final concentration) (LP8221, Bomei Biotechnology Co.,Ltd., Hefei, Anhui, China) was added to each well, followed by incubation at 37°C for 2 h. After incubation, 1% ZnAc (60 μL) (5970-45-6, Yinfeng Chemical Co. , Ltd. Weifang, Shandong, China), 10% TCA (60 μL), 0.2% (w/v) N,N-dimethyl-p-phenylenediamine (10 μL) (6606, Tianjin Chemical Reagent Institute Co. , Ltd., Tianjin, China) and 10% (w/v) ammonium ferric sulfate (10 μL) were added. The plate was incubated at room temperature in the dark for 15 min, followed by measuring the absorbance at 650 nm using the EnSpire Multimode Reader (PerkinElmer, United States). The H_2_S release amount was calculated based on the absorbance to evaluate the activity of CBS protein.

#### 3.1.3 Cellular thermal shift assay (CETSA)

CETSA was used to detect the binding efficiency of benserazide to CBS protein in tumor cells. Tumor cells treated with benserazide or vehicle (control group) were lysed using RIPA buffer and divided into seven groups. The protein extracts were heated at 37°C, 42°C, 47°C, 52°C, 57°C, 62°C, and 67°C for 20 min each before undergoing repeated freeze-thaw cycles to extract proteins. Western blot analysis was then performed to detect the expression of CBS protein and internal control protein at each heating temperature. The resulting thermal melting curves for the benserazide-treated and control groups were obtained by plotting the “CBS/Loading Control” *versus* temperature.

### 3.2 *In vitro* study

#### 3.2.1 MTS assay

The MTS assay was used to measure the cell viability *in vitro*. In this study, the KYSE450, A549, and HCT8 cells were cultured with test compounds in 96-well plates containing a final volume of 100 µL/well for 48 h. The MTS reagent (M8180, Solarbio Science &Technology Co.,Ltd., Beijing, China) was then added to each well and incubated with the cells for 4 h under standard culture conditions. The absorbance at 490 nm was measured using the EnSpire Multimode Reader (PerkinElmer, United States). These results were expressed as a percentage of control values, which represent the amount of formazan product produced by viable cells.

#### 3.2.2 EdU assay

The EdU assay was performed to determine the cell proliferation *in vitro*. In this study, the KYSE450, A549, and HCT8 cells were cultured with test compounds in 96-well plates for 24 h. The EdU assay kit (C10310-1, Guangzhou Ribbio Co., Ltd., Guangzhou, China) was then used to evaluate cell proliferation by measuring the number of EdU^+^ cells.

#### 3.2.3 Wound healing assay

The wound healing assay is a commonly used method to assess cell migration. In this study, the cells were seeded into six-well plates at a density of 4×10^5^ cells per well and a linear wound was created by pipetting with a 10 μL nozzle. The cells were then treated with test compounds and migration photos were taken at 0h and 24 h post-treatment. The gap closure rate was calculated by comparing the distance between the edges of the wound at 0h and 24 h.

#### 3.2.4 Transwell assay

The transwell assay is a method for assessing cell migration and invasion. In this study, the cells were seeded into the upper compartment of the transwell chamber at a density of 2×10^4^ cells per well in 200 µL of serum-free medium for the migration assay or with the matrix gel for the invasion assay. The lower chamber was filled with 500 µL of medium containing 10% serum. After 24 h of incubation, the cells on the upper surface of the membrane were fixed with 4% paraformaldehyde for 10 min and then stained with crystal violet for 30 min. The number of cells that had migrated or invaded through the membrane to the lower surface was counted under an inverted microscope.

#### 3.2.5 Tube formation assay

This experiment aims to investigate angiogenesis. Initially, 100 μL of matrix glue was added to each well of a 24-well plate and allowed to solidify for 30 min. Subsequently, 500 μL of conditioned medium and either HUVECs (2 × 10^5) or HLECs (2 × 10^5) were added to each well containing the matrix glue. The plate was then incubated at 37°C and 5% CO_2_ for 6–8 h. Finally, the capillaries formed were photographed under a 100× bright field microscope, and the number of capillaries in each well was counted.

#### 3.2.6 Western blot assay

The cells were lysed in a cell lysate containing 1% benzoyl fluoride (PMSF), and the protein concentration was determined using a BCA protein detection kit. Western blot analysis was then performed using a standard protocol. The protein samples were separated by electrophoresis on a gel plate and transferred to a PVDF membrane. After being closed at room temperature for 2 h, the membrane was incubated with primary antibodies overnight, washed three times with TBST buffer, and then incubated with secondary antibodies at room temperature for 2 h. Finally, the Enhanced Chemiluminescence (ECL) Assay Kit (PE0010, Solarbio Science &Technology Co.,Ltd., Beijing, China) was used to visualize the protein bands. The primary antibodies: CBS (14782, Cell Signaling Technology, Boston, United States), VEGF-C (2445, Cell Signaling Technology, Boston, United States), HIF-1α (20960-1-AP,Proteintech, Wuhan, Hubei, China), VEGF-A (19003-1-AP, Proteintech, Wuhan, Hubei, China), Notch1 (20687-1AP, Proteintech, Wuhan, Hubei, China), GAPDH (AG019, Bieyotime, Zhengzhou, Henan, China), SIRT1(CY5023, Abways, Shanghai, China), Hes1(CY5649, Abways, Shanghai, China), VEGF-D (CY10042,Abways, Shanghai, China).

#### 3.2.7 Methylene blue assay

Methylene blue assay was performed to determine the levels of H_2_S in tumor cells. L-cysteine (2 mM final concentration) and 5′-phospho pyridoxal 5′-phosphate (PLP) (5 mM final concentration) were added to the tumor cells cultured in a 6-well plate to promote H2S production. At the same time, 1% ZnAc (500 µL) was dropped onto filter paper attached to the lid of the 6-well plate to absorb the H2S released by the cells. After 24 h, the filter paper was placed into an eppendorf tube containing 0.2% (w/v) N, N-dimethyl-p-phenylenediamine (500 µL), 10% (w/v) ammonium ferric sulfate (50 µL), and 3 mL ddH2O. The tube was incubated at room temperature for 20 min before measuring the absorbance at 670 nm.

#### 3.2.8 The maleimide assay

Maleimide is a commonly used thiol-modifying reagent, and Cys residues are its reaction sites. Therefore, maleimide is used to detect the sulfhydryl modifications of proteins. Firstly, proteins are immunoprecipitated, and then maleimide (E1271-1G, Henan Puyingmei Biotechnology Co., Ltd., Zhengzhou, Henan, China) selectively labels unmodified and sulfonated cysteines. DTT (D1070-1, Solarbio Science &Technology Co.,Ltd., Beijing, China) is then used to reduce disulfides (-S-S-Mal). Finally, the samples are placed on polyacrylamide gels for scanning to detect fluorescently labeled proteins, and image analysis software is used for fluorescence quantification.

#### 3.2.9 ELISA

ELISA (Enzyme-Linked Immunosorbent Assay) was used to measure the concentration of proteins in a sample. In this case, VEGF-A, VEGF-C, and VEGF-D levels in the cell culture supernatant were detected using ELISA kits (ELK1129, ELK1194, ELK1195, ELK Biotechnology CO., LTD., Wuhan, China). The optical density (OD) was measured the EnSpire Multimode Reader (PerkinElmer, United States) at a wavelength of 450 nm. By comparing the OD values obtained for different samples or controls, we obtained the relative levels of VEGF-A, VEGF-C, and VEGF-D in the cell culture supernatant.

### 3.3 *In vivo* study

C57BL/6J mice (Four-week-old, weighing 16–18 g) were housed in sterile breeding cages, with six animals per cage, under constant temperature (approximately 22°C), humidity (45%–65%), a 12-h light/12-h dark cycle, minimal noise conditions, and provided with ample access to food, water, and sterile bedding. After a 1-week acclimation period in this environment, each mouse received a subcutaneous injection of 1.5×10^6^ AKR mouse esophageal cancer cells into the right hind paw pad to establish a lymph node metastasis model. The mice were then randomly divided into four groups (control group, benserazide treatment group, paclitaxel treatment group, benserazide plus paclitaxel treatment group), six mice per group. The treatment groups received daily intraperitoneal injections (benserazide 20 mg/kg, paclitaxel 5 mg/kg), while the control group received an equivalent volume of saline. During the treatment, pain and distress were minimized and alleviated through close monitoring of the mice’s behavior and weight changes, environmental enrichment, and humane endpoints. After 14 days, the mice were humanely sacrificed using cervical dislocation. Immediately after sacrifice, Lymph nodes and visceral organs such as the heart, liver, spleen, lungs, and kidneys were harvested from mice and fixed in 10% paraformaldehyde for HE staining. The experiment was conducted according to the methods described in reference (Liu et al., 2015). This experiment was approved by the Ethics Committee of Medical School of Henan University (HUSOM 2020-095, 25 March 2020).

### 3.4 Statistical analysis

The experiments were conducted with at least three repetitions. The data was presented as mean ± standard deviation (SD), and analyzed using GraphPad Prism eight software. For comparing paired data, the *t*-test is used to determine if there is a significant difference between the means of two groups. When comparing multiple groups of data, we first employ the Shapiro-Wilk test to check for normality. For data that are normally distributed, one-way Analysis of Variance (ANOVA) followed by Tukey’s multiple comparisons test is used, whereas for data that do not follow a normal distribution, such as those presented in Figure 7B (KYSE450 cells), Figure 7C (A549 cells), Figure 7E (Hes1 protein), Figure 8G (VEGF-A protein), Figure 8J (VEGF-A/D proteins), and Figure 9C, the Kruskal–Wallis H test is utilized to assess the significance of differences. Every possible comparison between the study groups was considered. *p* < 0.05 was considered statistically significant.

## 4 Results

### 4.1 CBS is involved in the progress of multiple cancers

**FIGURE 1:**
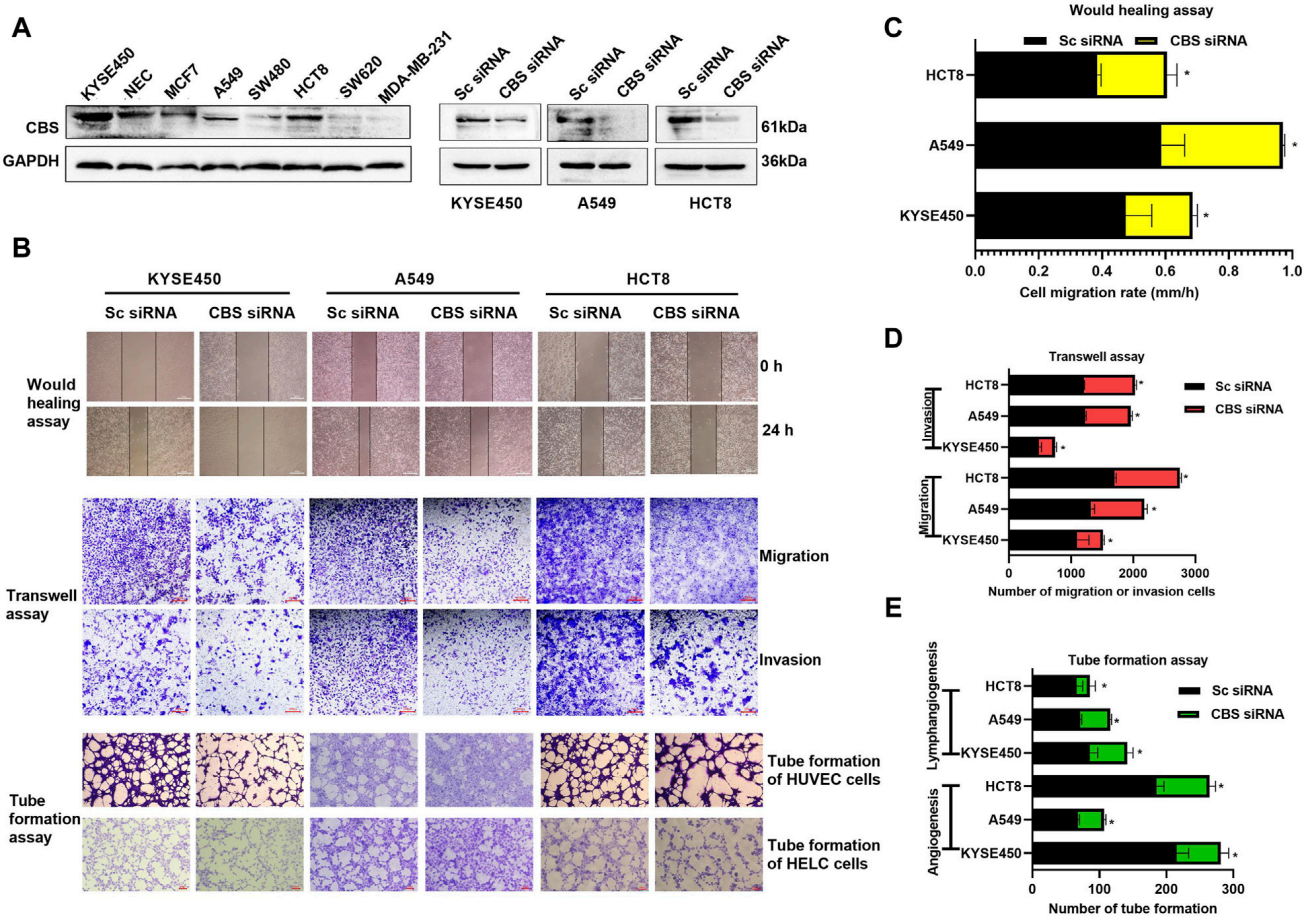
The impact of CBS expression on tumor cell growth and metastasis. **(A)** Expression levels of CBS protein in various cancer cells and validation of activity following CBS siRNA-mediated downregulation of CBS expression. **(B–E)** The effects of CBS downregulation on cancer cell migration, invasion, angiogenesis, and lymphangiogenesis. The scale of the images in the wound healing assay is 500 μm. The scale of the images in the transwell and tube formation assays is 200 μm. All data are expressed as mean ± standard deviation. ∗P < 0.05. CBS, cystathionine beta-synthase.

Previous research has indicated that Cystathionine β-synthase (CBS) is highly expressed and contributes to the progression of various types of cancer. Here, we further demonstrated that CBS is highly expressed in KYSE450, NEC, MCF7, A549 and HCT8 cells (Figure 1A) and downregulation of CBS (Figure 1A) has been shown to inhibit tumor cell migration and invasion, as well as suppress angiogenesis and lymphangiogenesis (Figures 1B–E) in KYSE450, A549 and HCT8 cells. This suggests that CBS plays an important role in the progression of various types of cancer.

### 4.2 Benserazide inhibits CBS by forming a stable complex with CBS protein

**FIGURE 2:**
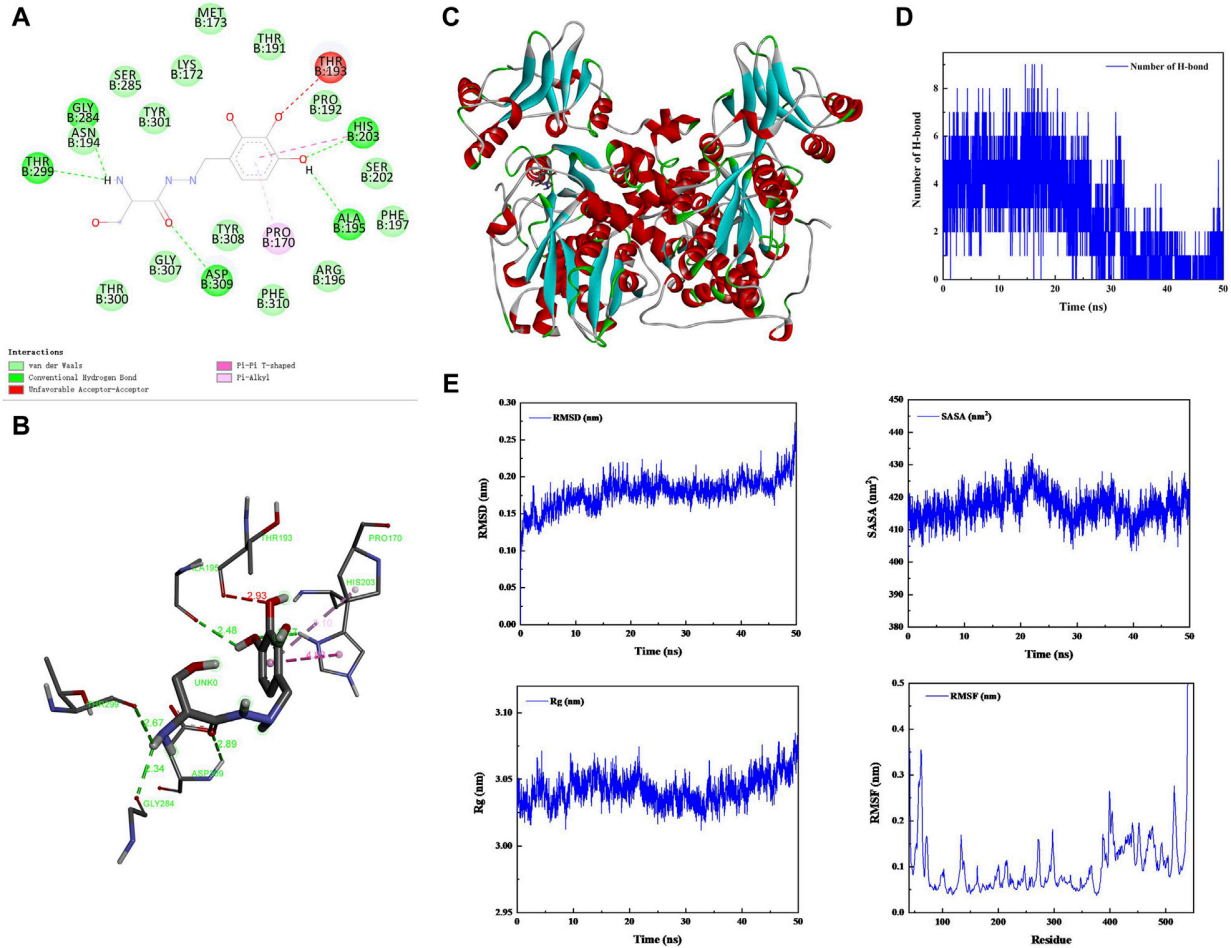
Analysis of the bingding mode of Benserazide with CBS protein. **(A–C)** 2D and 3D images of the molecular docking between Benserazide and CBS protein. **(D)** The curve showing the change in the number of hydrogen bonds over time during the simulation process of the protein-ligand complex. **(E)** The curves illustrating the changes in the protein’s RMSD, SASA, Rg, and RMSF over time during the simulation process of the protein-ligand complex. RMSD, Root Mean Square Deviation; SASA, Solvent-accessible Surface Areas; Rg, rotation radius; RMSF, Root Mean Square Fluctuation.

Benserazide is a peripheral dopamine decarboxylase inhibitor that has been shown to have CBS protease inhibitory activity. To verify the function of benserazide, we first analyzed its binding mode with CBS protein using molecular docking. It is found that the binding free energy of benserazide and CBS protein is −6.9 kcal/mol and CBS protein contains several amino acid residues that are capable of forming hydrogen bonds with benserazide, namely, ALA195, THR299, ASN194, HIS203, and ASP309. Furthermore, HIS203 is also able to engage in π-π interactions with benserazide. Additionally, PRO170 of the protein can form Pi-Alkyl interactions with benserazide. The binding mode of benserazide to CBS protein is shown in Figures 2A–C. Subsequently, molecular dynamics analysis was employed to assess the stability of the conformation. The analysis of Hydrogen bond, root mean square deviation (RMSD), solvent-accessible surface areas (SASA), rotation radius (Rg) and Root Mean Square Fluctuation (RMSF) showed that benserazide and CBS protein are able to bind stably (Figures 2D, E). To better explain the interaction energy between protein and ligand, we used the gmx_mmpbsa (https://jerkwin.github.io/gmxtool/) script to determine all binding energies of protein-ligand complexes during the equilibrium stage. The results showed that in the protein-ligand complex system, the total binding free energy between protein and small molecule is −98.241 kJ/mol (Table 1), indicating a stable binding between protein and small molecule.

**TABLE 1:** Analysis of protein ligand MMPBSA.

Energy	Complex
Van der Waals Energy (KJ/mol)	−121.873
Electrostatic energy (kJ/mol)	−8.680
Polar solvation energy (KJ/mol)	49.798
Nonpolar solvation Energy (KJ/mol)	−17.486
Total Binding Energy (KJ/mol)	−98.241

**FIGURE 3:**
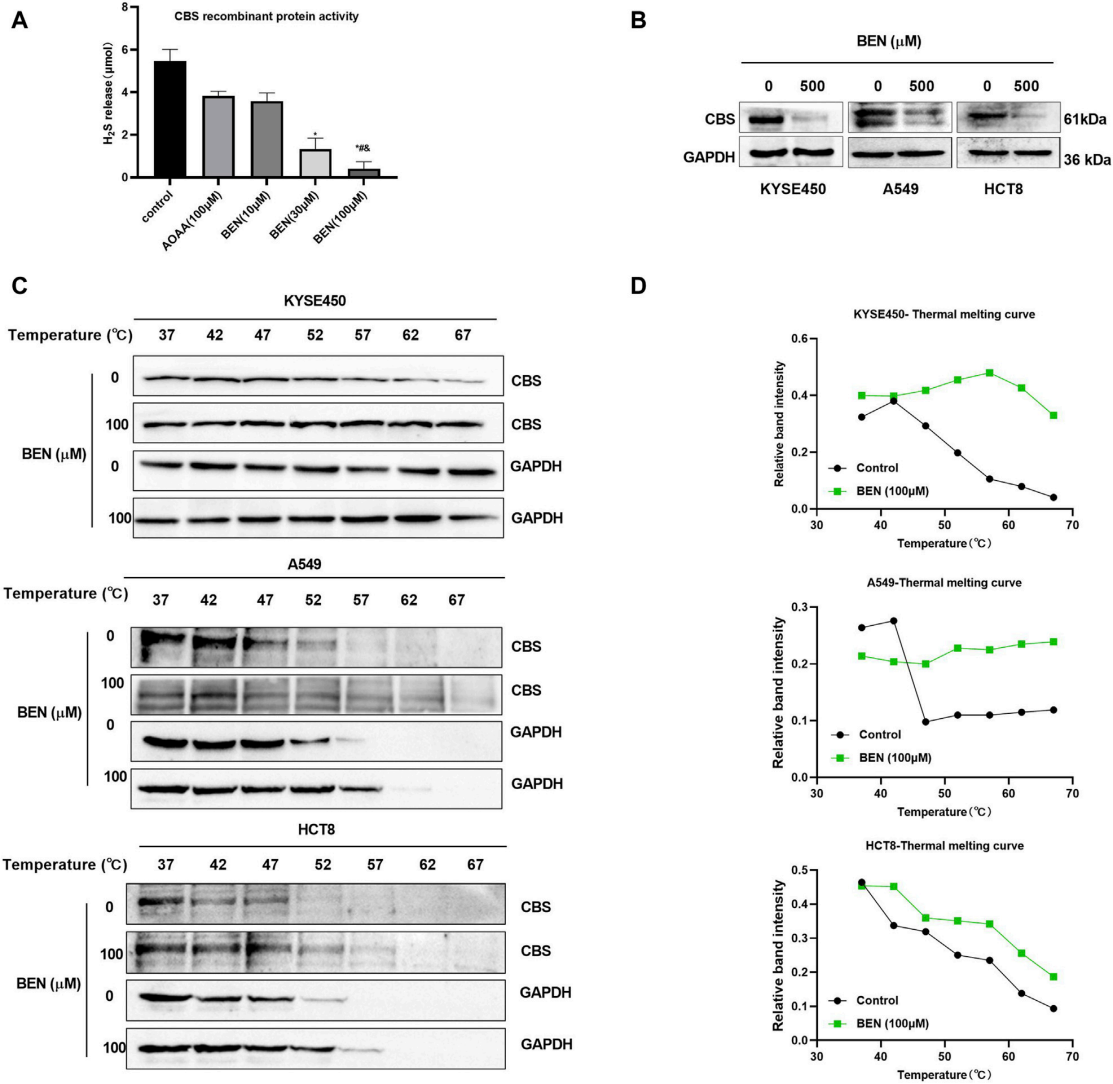
Benserazide binds to and blocks the activity of CBS in cancer cells. **(A)** Detection of H_2_S release from CBS recombinant protein after drug treatment using the methylene blue method. All data are expressed as mean ± standard deviation. ^∗^ *p* < 0.05 vs. control group. ^#^ *p* < 0.05 vs. AOAA group. ^&^ *p* < 0.05 vs. BEN (10 μM) group. **(B)** The effect of benserazide on CBS expression in cancer cells. **(C, D)** CETSA to assess the binding capacity of Benserazide with CBS in tumor cells. BEN, Benserazide; CBS, cystathionine beta-synthase; CETSA, Cellular Thermal Shift Assay.

In addition, the inhibitory effect of benzserazide on CBS protein was further investigated. It was discovered that the release of H_2_S in the 100 μM benserazide-treated group was merely 7.5% of that in the negative control, and significantly lower than that in the positive control AOAA-treated group (Figure 3A). Furthermore, our observations revealed that benserazide had an inhibitory effect on the expression of CBS protein in cancer cells, as shown in Figure 3B. Additionally, CETSA assay was performed to detect whether benserazide can bind to CBS in cancer cells and it was found that the amount of undegraded CBS protein increased in the presence of benserazide, and the thermal melting curve of the complex protein shifted to the right at the denaturation temperature of 52°C–67°C (Figures 3C, D), indicating that benzserazide can bind to CBS protein in cancer cells. The findings suggest that Benserazide can inhibit the activity of CBS protein by forming a stable complex with it.

### 4.3 Benserazide inhibits the growth and metastasis of tumor cells

**FIGURE 4:**
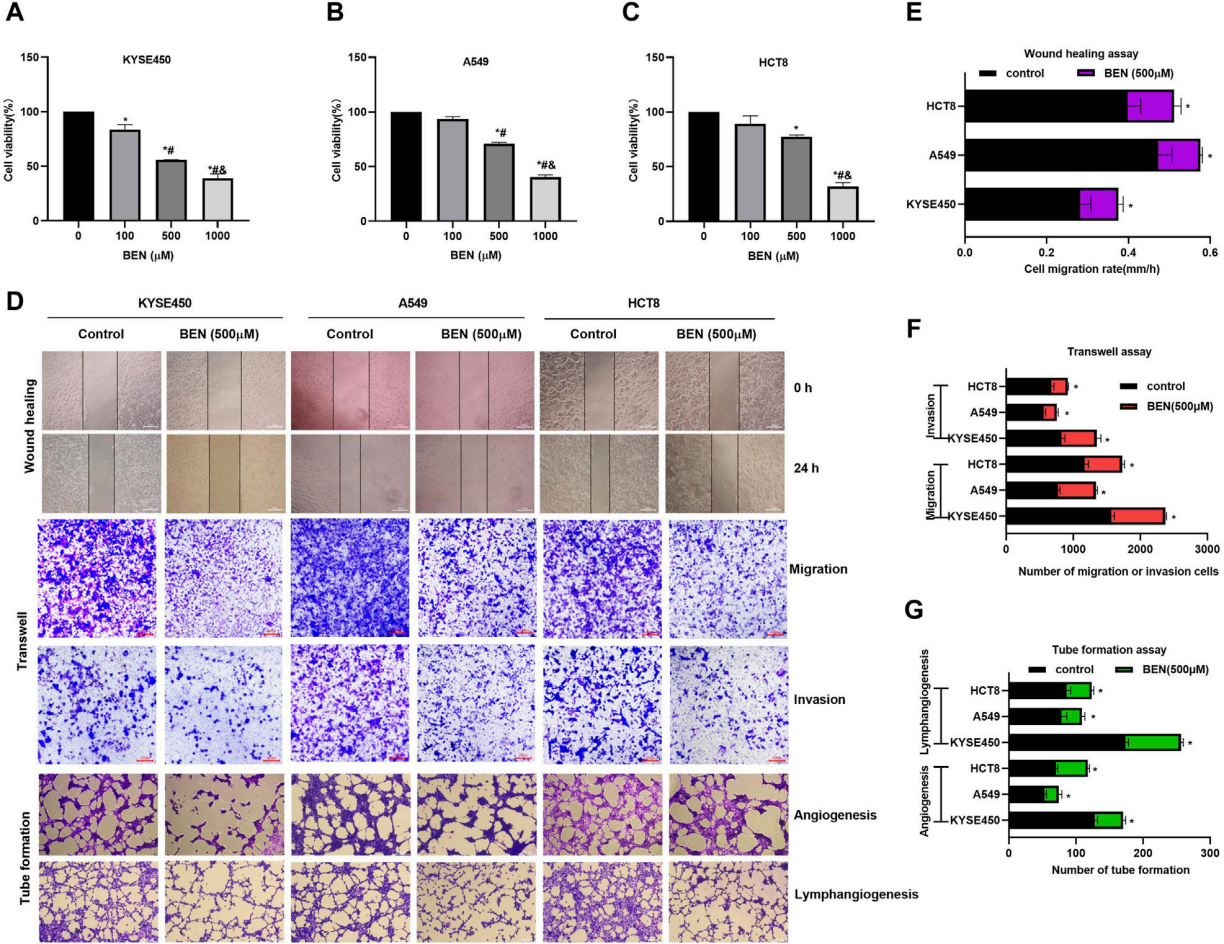
Benserazide inhibits the growth and metastasis of tumor cells. **(A–C)** The impact of Benserazide on the viability of KYSE450, A549 and HCT8 cells. All data are expressed as mean ± standard deviation. ∗*p* < 0.05 vs. BEN (0 μM) group. #*p* < 0.05 vs. BEN (100 μM) group. & *p* < 0.05 vs. BEN (50 μM) group. **(D–G)** The effect of benserazide on the migration, invasion, angiogenesis, and lymphangiogenesis of KYSE450, A549 and HCT8 cells. The scale of the images in the wound healing assay is 500 μm. The scale of the images in the transwell and tube formation assays is 200 μm. All data are expressed as mean ± standard deviation. ∗*p* < 0.05 vs. control group. BEN, Benserazide.

In order to investigate the role of benserazide in cancer cells, we conducted a series of experiments. Our results from the MTT assay showed that benserazide inhibited tumor cell growth in a concentration-dependent manner (Figures 4A–C). Furthermore, we examined the effect of benserazide on tumor cell metastasis *in vitro* using a wound healing assay and a transwell assay. The findings suggested that benserazide could impede the migratory and invasive abilities of cancer cells (Figures 4D–F). In addition, our tube formation experiment revealed that benserazide significantly inhibited tumor cell angiogenesis and lymphangiogenesis when compared to the control group (Figures 4D, G). These findings suggest that benserazide possesses potential antitumor activity.

### 4.4 Benserazide enhances the antitumor activity of paclitaxel by modulating the SIRT1 pathway

**FIGURE 5:**
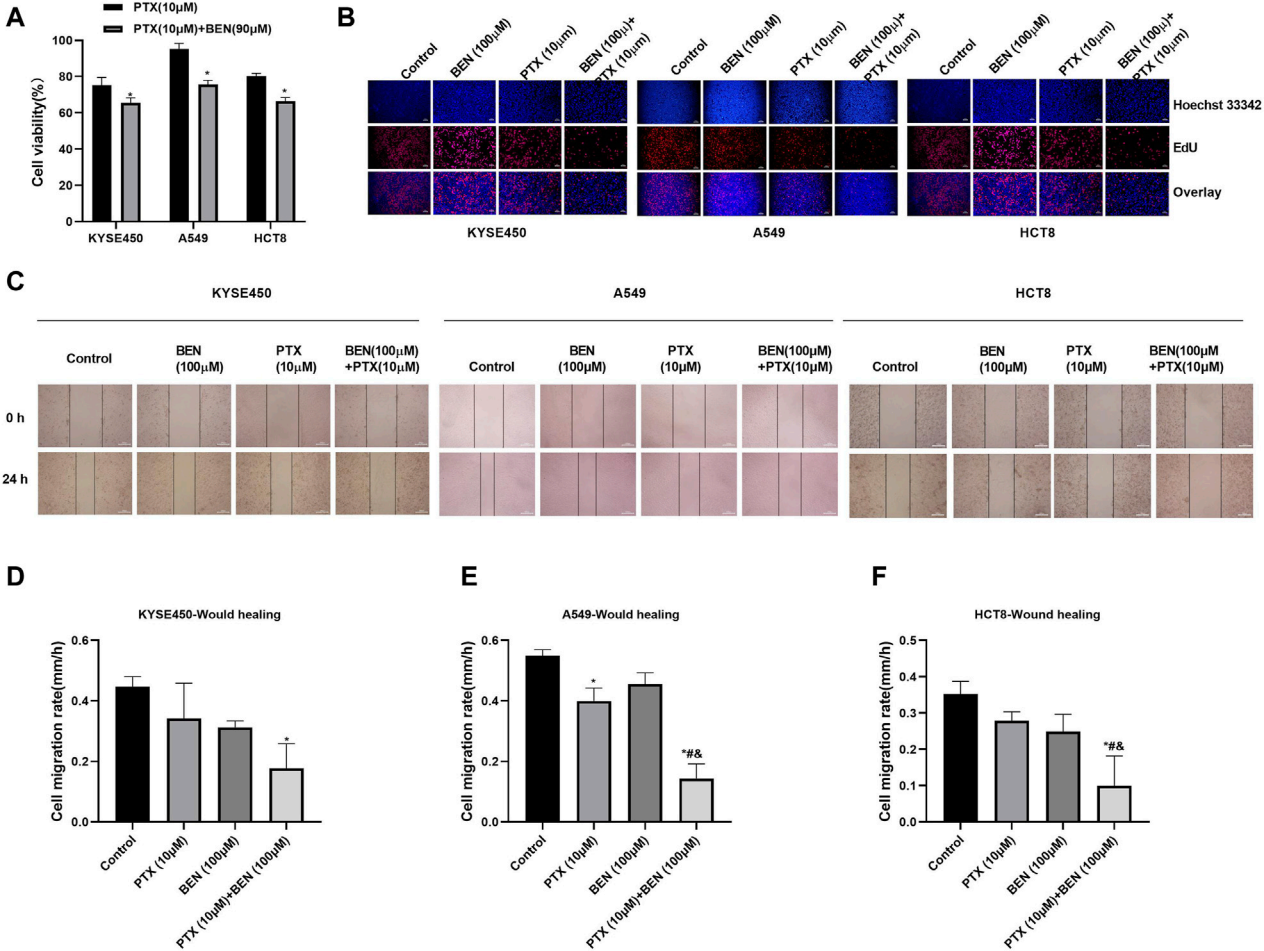
Benserazide in combination with Paclitaxel suppresses the proliferation of tumor cells. **(A)** The effect of Benserazide in combination with Paclitaxel on cell viability of KYSE450, A549 and HCT8 cells. All data are expressed as mean ± standard deviation. ^∗^ *p* < 0.05 vs. control group. **(B)** The effect of Benserazide in combination with Paclitaxel on cell proliferation of KYSE450, A549 and HCT8 cells. The scale of the images in the EdU assay is 200 μm. **(C–F)** The effect of Benserazide in combination with Paclitaxel on the migration of KYSE450, A549 and HCT8 cells. The scale of the images in the wound healing assay is 500 μm. All data are expressed as mean ± standard deviation. ^∗^ *p* < 0.05 vs. control group. ^#^ *p* < 0.05 vs. PTX group. ^&^ *p* < 0.05 vs. BEN group. BEN, Benserazide; PTX, Paclitaxel; EdU, 5-Ethynyl-2′-deoxyuridine.

**FIGURE 6:**
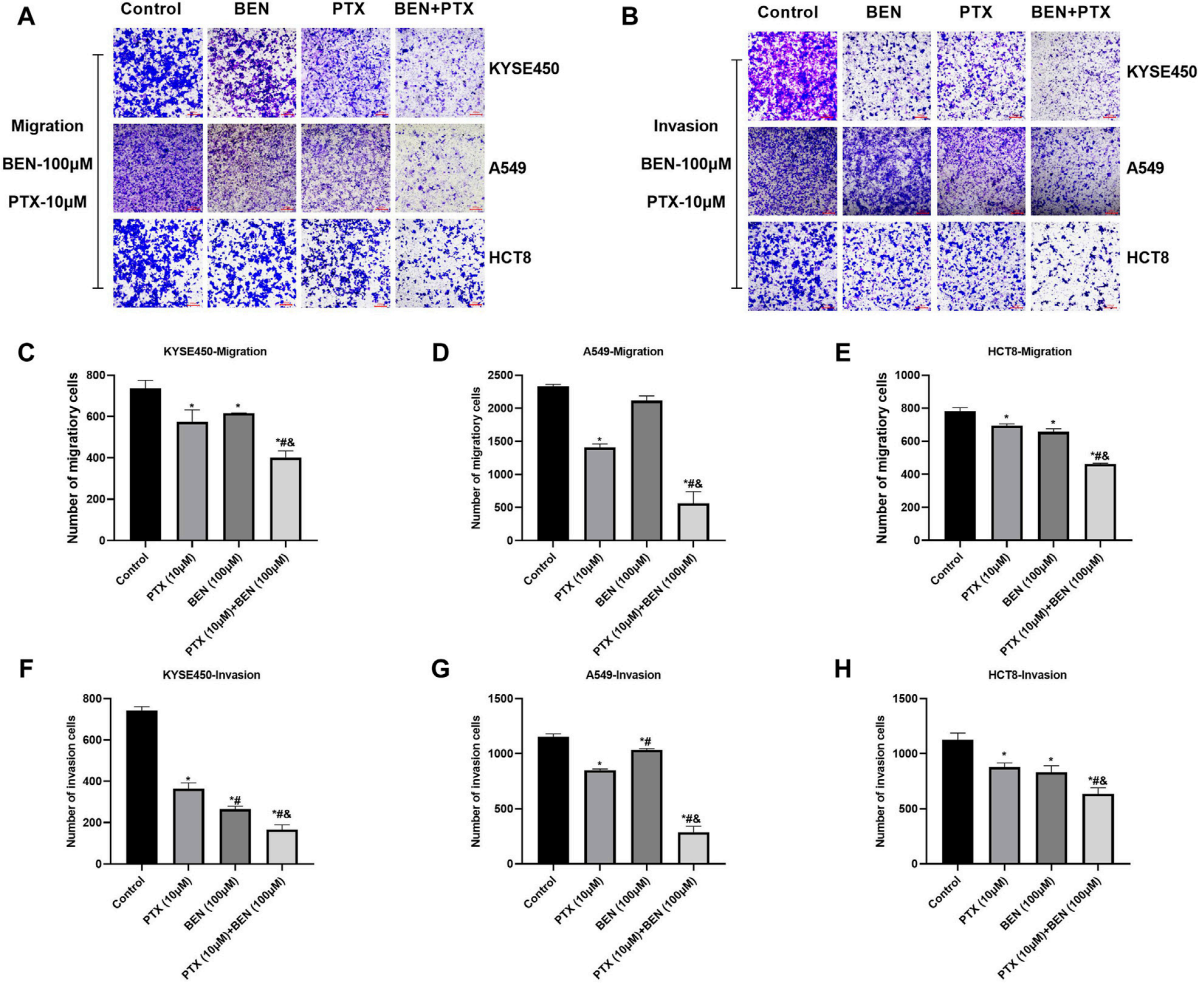
Benserazide in combination with Paclitaxel inhibits the migration and invasion of tumor cells. **(A, B)** The effect of Benserazide in combination with Paclitaxel on the migration and invasion of KYSE450, A549 and HCT8 cells. The scale of the images in the transwell assay is 200 μm. **(C–H)** The statistical analysis of migratory or invasion cells. All data are expressed as mean ± standard deviation. ^∗^ *p* < 0.05 vs. control group. ^#^ *p* < 0.05 vs. PTX group. ^&^ *p* < 0.05 vs. BEN group. BEN, Benserazide; PTX, Paclitaxel.

In order to find a new therapeutic strategy that can increase the sensitivity of tumor therapy while reducing the dose of traditional chemotherapy drugs, we investigated the sensitization effect of benserazide on paclitaxel. By administering a nontoxic dose of benserazide (100 μM), we found that the combined administration of benserazide and paclitaxel could significantly reduce the viability of tumor cells (Figure 5A) and inhibit tumor cell proliferation (Figure 5B), migration and invasion (Figures 5C–F, 6) more effectively than paclitaxel alone. These results suggest that the addition of benserazide can enhance the sensitivity of chemotherapy drug, thereby increasing the efficiency of tumor therapy.

**FIGURE 7:**
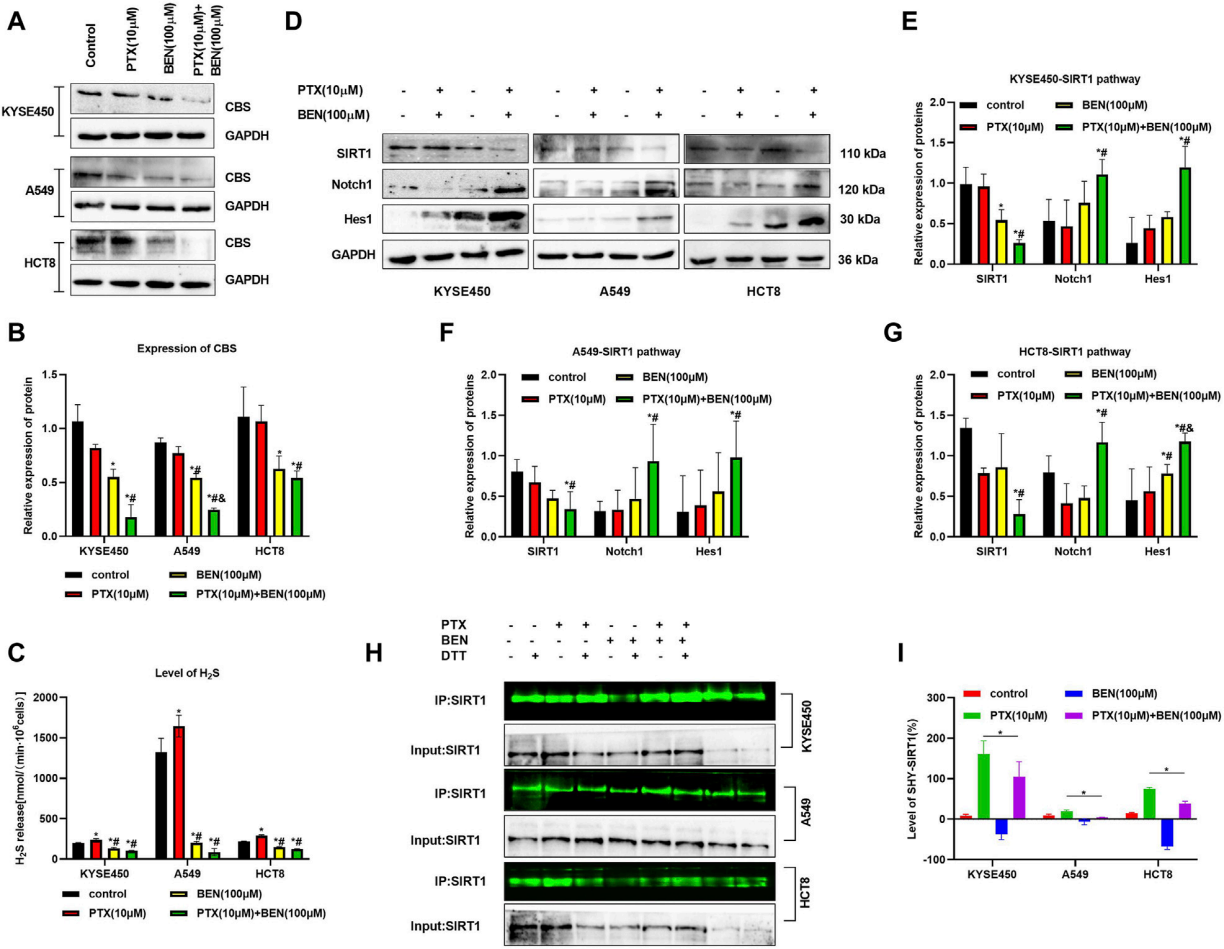
Benserazide in combination with Paclitaxel prevents the S-sulfhydration of SIRT1 protein. **(A, B)** Benserazide in combination with Paclitaxel reduces the level of CBS protein in KYSE450, A549 and HCT8 cells. **(C)** Benserazide in combination with Paclitaxel inhibits the production of H2S in KYSE450, A549 and HCT8 cells. **(D–G)** Benserazide in combination with Paclitaxel downregulates the SIRT1 signal pathway in KYSE450, A549 and HCT8 cells. **(H–I)** Benserazide in combination with Paclitaxel decreases the S-sulfhydration level of SIRT1 protein in KYSE450, A549 and HCT8 cells. All data are expressed as mean ± standard deviation. The data pertaining to panel B (KYSE450 cells), panel C (A549 cells), and panel E (Hes1 protein) do not adhere to a normal distribution. ^∗^ *p* < 0.05 vs. control group. ^#^ *p* < 0.05 vs. PTX group. ^&^ *p* < 0.05 vs. BEN group. BEN, Benserazide; PTX, Paclitaxel; SIRT1, Sirtuin 1; Notch1, Notch homolog 1; Hes1, Hairy and Enhancer of Split 1.

SIRT1 upregulation has already been demonstrated in various cancer cells, such as esophageal squamous cell carcinoma (ESCC), lung cancer, and colon cancer. In addition, some studies have found that the activation of Notch1/Hes1 signaling pathway, as a downstream pathway of SIRT1, can induce apoptosis in esophageal squamous cell carcinoma and colon cancer cells, as well as inhibit their lymph node metastasis. Furthermore, research has demonstrated that H_2_S can enhance the expression of SIRT1 by facilitating its S-sulfhydration. In this study, we examined the impact of benserazide on the SIRT1 pathway to elucidate the mechanism by which CBS inhibitor benserazide enhances paclitaxel’s antitumor activity. Our investigation found that benserazide alone and in combination with paclitaxel significantly decreased the level of CBS/H_2_S system in KYSE450, A549, and HCT8 cells (Figures 7A–C). Western blot results showed that the combination of benserazide and paclitaxel significantly inhibited the expression of SIRT1 and caused an increase in the levels of SIRT1 downstream proteins Notch1 and Hes1 in KYSE450, A549, and HCT8 cells compared to paclitaxel alone (Figures 7D–G). Furthermore, maleimide assay results showed that the combination of benserazide and paclitaxel significantly reduced the levels of S-sulfhydration of SIRT1 in KYSE450, A549, and HCT8 cells compared to paclitaxel alone (Figures 7H, I). These findings suggest that benserazide inhibits SIRT1 signaling pathway by preventing the S-sulfhydration of SIRT1, which in turn potentiates the antitumor effects of paclitaxel.

### 4.5 Benserazide combined with paclitaxel inhibits angiogenesis and lymphangiogenesis through suppressing the HIF1-1α/VEGF pathway

**FIGURE 8:**
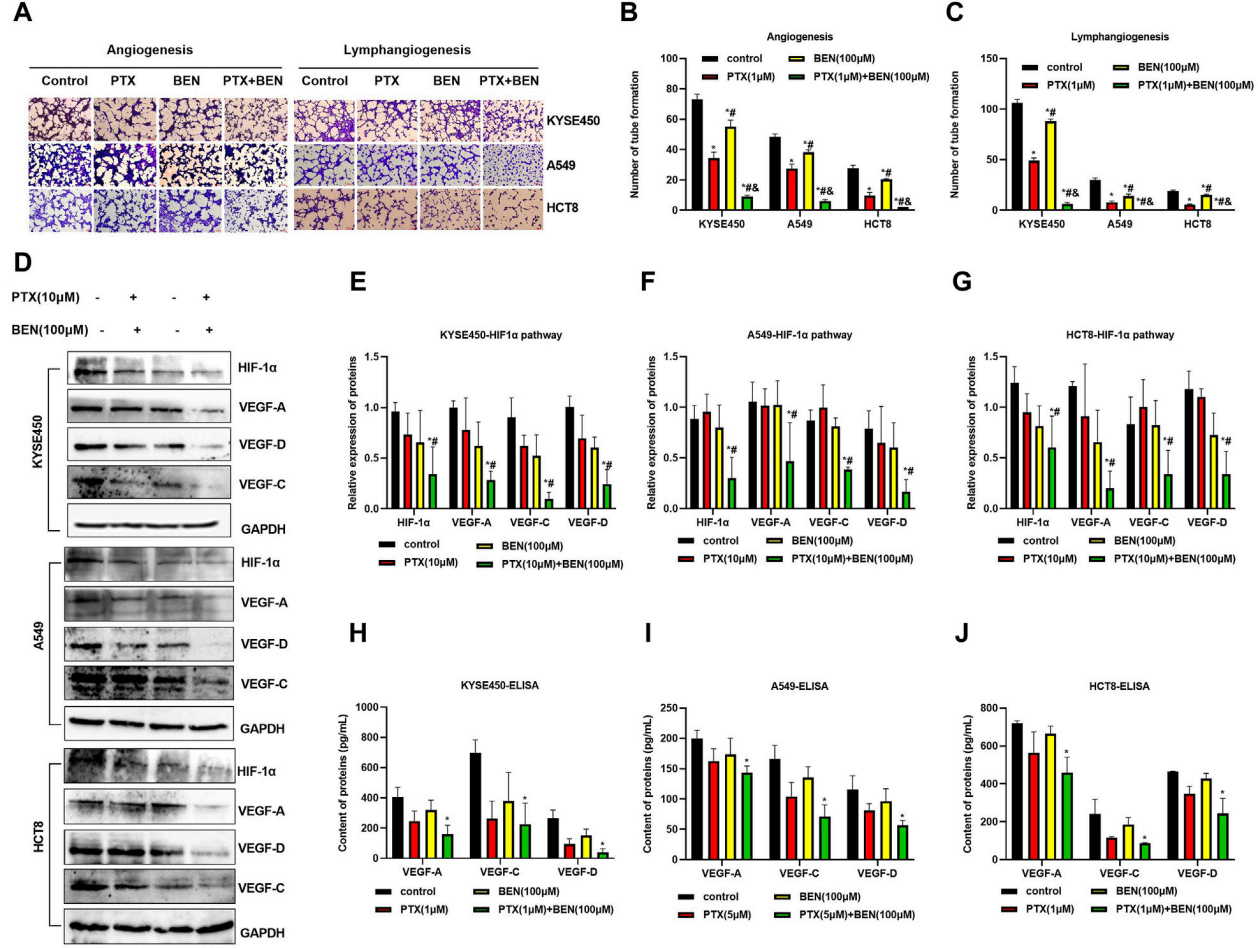
Benserazide combined with paclitaxel inhibits angiogenesis and lymphangiogenesis through suppressing the HIF1-1α/VEGF pathway. **(A–C)** The impact of Benserazide combined with Paclitaxel on angiogenesis and lymphangiogenesis induced by KYSE450, A549 and HCT8 cells. The scale of the images in the tube formation assays is 200 μm. **(D–G)** The effect of Benserazide combined with Paclitaxel on the HIF1-1α/VEGF pathway in KYSE450, A549 and HCT8 cells. **(H–J)** ELISA assay to detect intracellular VEGF-A, VEGF-C and VEGF-D levels. All data are expressed as mean ± standard deviation. The data pertaining to panel G (VEGF-A protein) & panel J (VEGF-A/D proteins) do not adhere to a normal distribution. ^∗^ *p* < 0.05 vs. control group. ^#^ *p* < 0.05 vs. PTX group. ^&^ *p* < 0.05 vs. BEN group. BEN, Benserazide; PTX, Paclitaxel; HIF-1α, Hypoxia-induced factor 1α; VEGF, Vascular Endothelial Growth Factor.

Angiogenesis and lyrmphangiogenesis are essential processes for the metastasis of tumor cells. In this study, we investigated the effect of benserazide combined with paclitaxel on angiogenesis and lymphangiogenesis of tumor cells *in vitro*. Our results from the tube formation experiment showed that the combined administration significantly inhibited the ability of tumor cells to induce neovascularization through HUVECs and HLECs (Figures 8A–C).

The hypoxia-induced factor HIF-1α and VEGF play a crucial role in tumor angiogenesis and lymphangiogenesis. Specifically, HIF-1α can increase the expression of VEGF, which in turn promotes both angiogenesis and lymphangiogenesis. In this study, we observed that benserazide combined with paclitaxel induced a more significant downregulation of HIF-1α expression levels in KYSE450, A549, and HCT8 cells compared to paclitaxel alone (Figures 8D–G). Additionally, we found that the expression of VEGF-A, VEGF-C, and VEGF-D proteins was significantly reduced in the combination treatment group (Figures 8D–G). We further used ELISA to detect the levels of VEGF-A, VEGF-C, and VEGF-D and observed that the combination treatment group had lower levels of all three proteins (Figures 8H–J). These findings indicate that benserazide enhances the inhibitory effect of paclitaxel on tumor cell angiogenesis and lymphangiogenesis via the HIF-1α/VEGF pathway.

### 4.6 Benserazide combined with paclitaxel inhibits lymph node metastasis of mouse transplanted tumors

**FIGURE 9:**
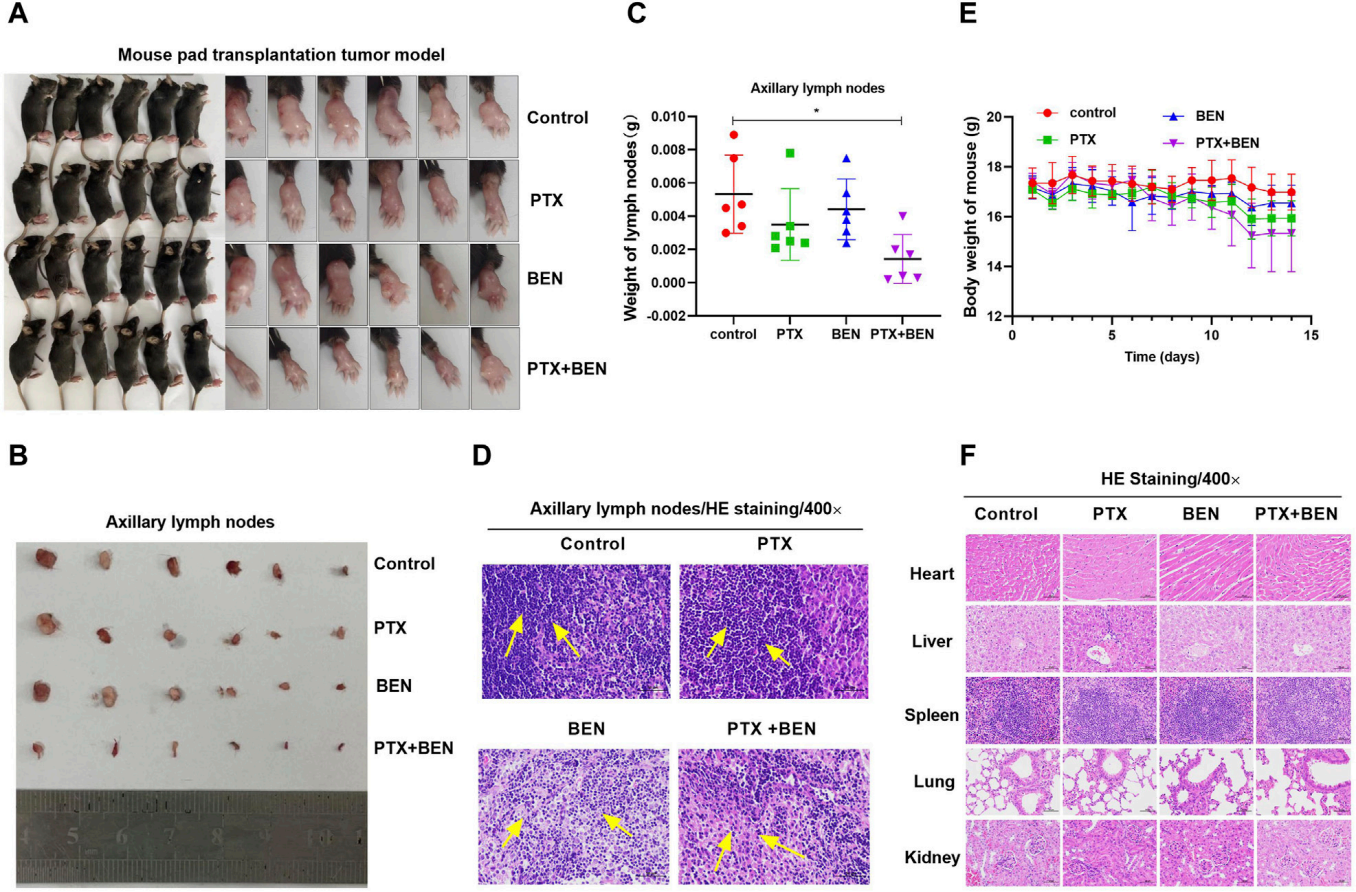
Benserazide combined with paclitaxel inhibits lymph node metastasis of mouse transplanted tumors. (A) Mouse pad transplantation tumor model. **(B, C)** The effect of Benserazide combined with Paclitaxel on volume and weight of axillary lymph nodes. **(D)** H&E staining to detect the tumor cell infiltration in the lymph nodes. The arrows indicate the infiltration of inflammatory or tumor cells. **(E)** The effect of Benserazide combined with Paclitaxel on the body weight of mice. **(F)** H&E staining to detect the morphology changes of heart, liver, spleen, lung, and kidney of mice. All data are expressed as mean ± standard deviation. The data pertaining to panel C do not adhere to a normal distribution. ^∗^ *p* < 0.05 vs. control group. BEN, Benserazide; PTX, Paclitaxel.

Based on the aforementioned results that benserazide in combination with paclitaxel has an inhibitory effect on angiogenesis and lymphangiogenesis, we aimed to further investigate their *in vivo* activity when used together. To achieve this, we established a mouse pad transplantation tumor model to study their impact on lymph node metastasis (Figure 9A). Our observations revealed that the combined administration of benserazide and paclitaxel significantly reduced the volume and weight of axillary lymph nodes in comparison to the control group and single administration group (Figures 9B, C). Furthermore, H&E staining results demonstrated that tumor cell infiltration in the lymph nodes was also significantly lower in the combined administration group compared to the single administration group (Figure 9D). Additionally, the body weight of mouse and the morphology of heart, liver, spleen, lung, and kidney of mice had no significant differences in the combined administration group when compared to the control group and single administration group (Figures 9E, F). These findings suggest that benserazide in combination with paclitaxel has a potent inhibitory effect on tumor lymph node metastasis *in vivo*.

## 5 Discussion

As an advanced or metastatic tumor treatment, chemotherapy is the primary option. Paclitaxel-based regimens are the most commonly used chemotherapy treatments. Recent clinical trials have focused on adding targeted therapies to chemotherapy. In this study, we evaluated the therapeutic potential of benserazide, a targeting CBS inhibitor, in combination with paclitaxel to inhibit tumor growth and understand the mechanism of action of these drugs.

Firstly, we verified whether benserazide targets and inhibits the CBS protein. Molecular docking results showed that the binding free energy of benserazide and CBS protein is −6.9 kcal/mol, and CBS protein contains amino acid residues capable of forming hydrogen bonds, π-π interactions, and Pi-Alkyl interactions with benserazide. Additionally, it was discovered that benserazide significantly inhibits the activity of recombinant CBS protein with a stronger inhibition than AOAA. Furthermore, CETSA assay found that the presence of benserazide increased the amount of undegraded CBS protein and caused the thermal melting curve of the complex protein to shift to the right at the denaturation temperature of 52°C–67°C, indicating that benserazide can bind to CBS protein in cancer cells. Our observations also revealed that benserazide had an inhibitory effect on the expression of CBS protein in cancer cells. The findings suggest that Benserazide Inhibits CBS by Forming a Stable Complex with CBS Protein.

Next, we investigated the sensitization effect of benserazide on paclitaxel *in vitro* and *in vivo* to find a new therapeutic strategy that can increase the sensitivity of tumor therapy. We found that the combined administration of a nontoxic dose of benserazide (inhibitory rate of cells <10%) and paclitaxel could significantly reduce the viability of tumor cells and inhibit tumor cell proliferation, migration, invasion, angiogenesis, and lymphangiogenesis more effectively than paclitaxel alone *in vitro*. In addition, Benserazide combined with paclitaxel inhibits lymph node metastasis of mouse transplanted tumors. These results suggest that the addition of benserazide can enhance the sensitivity of chemotherapy drugs, thereby increasing the efficiency of tumor therapy.

To further explore the mechanism of benserazide enhancing paclitaxel anticancer activity, we investigated the effect of the combination of benserazide and paclitaxel on SIRT1 and HIF-1a signaling pathways.

Sirtuin-1 (SIRT1) is a class-III histone deacetylase (HDAC), an NAD + -dependent enzyme deeply involved in gene regulation, genome stability maintenance, apoptosis, autophagy, senescence, proliferation, aging, and tumorigenesis (Alves-Fernandes and Jasiulionis, 2019). SIRT1 upregulation has already been demonstrated in some cancer cells, such as esophageal squamous cell carcinoma (ESCC), lung cancer, and colon cancer (Hong and Pyo, 2018; Ma et al., 2018; Guo et al., 2021; Otsuka et al., 2022). Furthermore, increased expression of SIRT1 has been found to confer resistance to cisplatin in cancer cells (Cao et al., 2015; Wang et al., 2022; Sun et al., 2023). Moreover, research has demonstrated that H_2_S can enhance the expression of SIRT1 by facilitating its S-sulfhydration (Wu et al., 2023), which involves altering the Cys residues on the target protein to convert the sulfhydryl group (R-SH) into a persulfide group (R-SSH). In this study, we found that the combination of benserazide and paclitaxel significantly inhibited the S-sulfhydration of SIRT1 protein and the expression of SIRT1 protein, consequently activating downstream Notch1/Hes1 signaling pathway in KYSE450, A549, and HCT8 cells.

The hypoxia-induced factor HIF-1α and VEGF play a crucial role in tumor angiogenesis and lymphangiogenesis (Alam et al., 2023; Song et al., 2023). Specifically, HIF-1α can increase the expression of VEGF, which in turn promotes both angiogenesis and lymphangiogenesis. In this study, we observed that benserazide combined with paclitaxel induced a more significant downregulation of HIF-1α, VEGF-A, VEGF-C, and VEGF-D proteins expression levels in KYSE450, A549, and HCT8 cells compared to paclitaxel alone. The results indicated that Benserazide enhances the anticancer effects of paclitaxel via inhibiting the S-sulfhydration of SIRT1 and enhancing the anti-angiogenic effect of paclitaxel through downregulation of HIF-1α/VEGF signaling pathway proteins expression.

**FIGURE 10:**
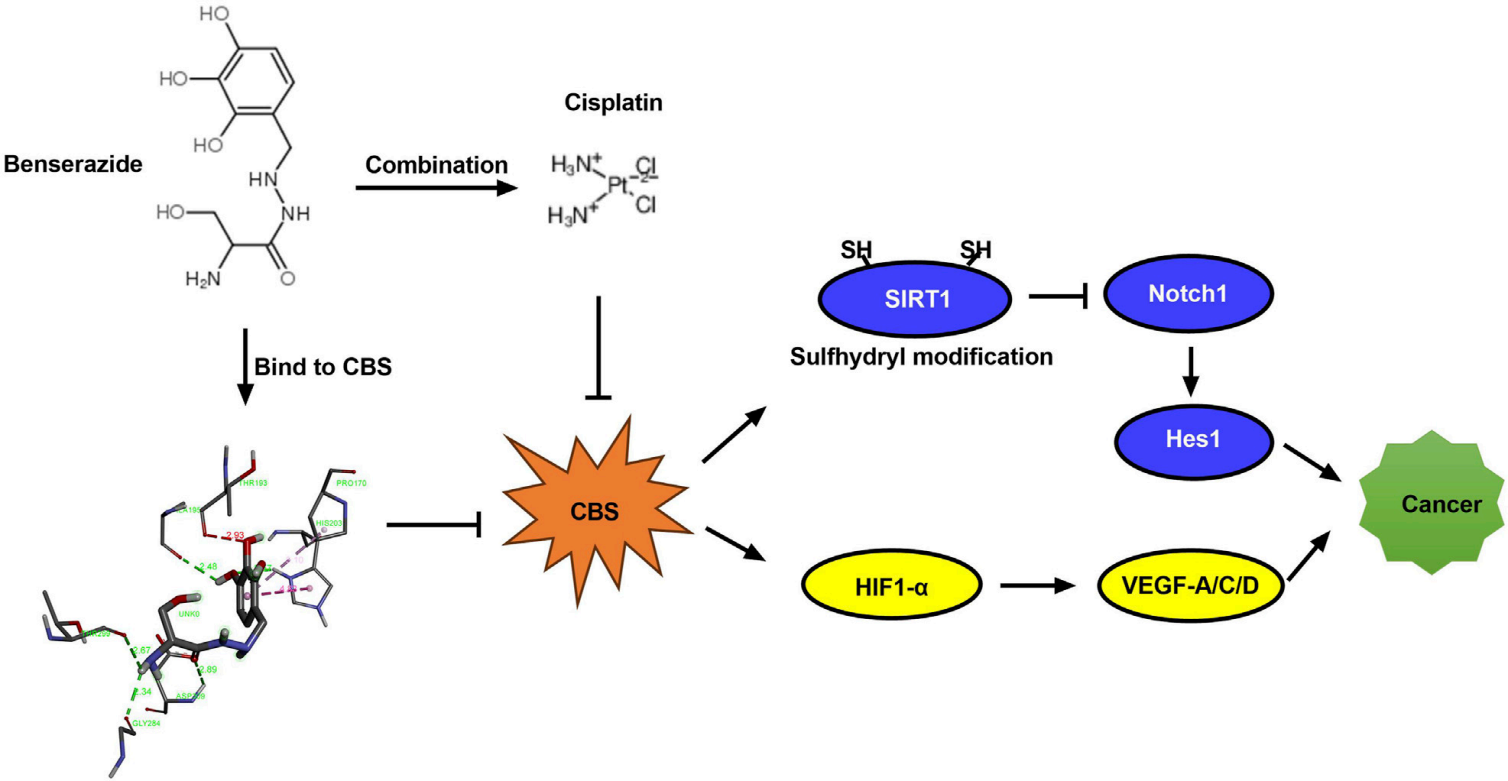
Diagram illustrating how benserazide augments the tumoricidal effect of paclitaxel. CBS, cystathionine beta-synthase; SIRT1, Sirtuin 1; Notch1, Notch homolog 1; Hes1, Hairy and Enhancer of Split 1; HIF-1α, Hypoxia-induced factor 1α; VEGF, Vascular Endothelial Growth Factor.

This study suggests that benserazide may have potential as a chemosensitizer in cancer treatment by enhancing the anti-cancer effect of paclitaxel through the inhibition of S-sulfhydration of SIRT1 and enhancing the anti-angiogenic effect of paclitaxel through downregulation of HIF-1α/VEGF signaling pathway proteins expression, as shown in Figure 10. This finding provides a new insight into the development of combination therapy for cancer treatment. In subsequent research, we will employ animal xenograft models by transplanting human cancer cells into immunodeficient mice to observe the impact of the drugs on the growth of the transplanted tumors. Additionally, we will assess the *in vivo* anticancer activity by measuring pharmacodynamic parameters, such as drug concentration, bioavailability, and half-life. It is hoped that benserazide may find application in the field of oncology in the future.

## Data Availability

The original contributions presented in the study are included in the article/Supplementary material, further inquiries can be directed to the corresponding authors.
